# Genomic surveillance, evolution and global transmission of SARS-CoV-2 during 2019–2022

**DOI:** 10.1371/journal.pone.0271074

**Published:** 2022-08-01

**Authors:** Nadim Sharif, Khalid J. Alzahrani, Shamsun Nahar Ahmed, Afsana Khan, Hamsa Jameel Banjer, Fuad M. Alzahrani, Anowar Khasru Parvez, Shuvra Kanti Dey

**Affiliations:** 1 Department of Microbiology, Jahangirnagar University, Savar, Dhaka, Bangladesh; 2 Department of Clinical Laboratories Sciences, College of Applied Medical Sciences, Taif University, Taif, Saudi Arabia; 3 Department of Statistics, Jahangirnagar University, Savar, Dhaka, Bangladesh; Translational Health Science & Technology Institute, INDIA

## Abstract

In spite of the availability of vaccine, the health burden associated with the COVID-19 pandemic continues to increase. An estimated 5 million people have died with SARS-CoV-2 infection. Analysis of evolution and genomic diversity can provide sufficient information to reduce the health burden of the pandemic. This study focused to conduct worldwide genomic surveillance. About 7.6 million genomic data were analyzed during 2019 to 2022. Multiple sequence alignment was conducted by using maximum likelihood method. Clade GK (52%) was the most predominant followed by GRY (12%), GRA (11%), GR (8%), GH (7%), G (6%), GV (3%), and O (1%), respectively. VOC Delta (66%) was the most prevalent variant followed by VOC Alpha (18%), VOC Omicron (13%), VOC Gamma (2%) and VOC Beta (1%), respectively. The frequency of point mutations including E484K, N501Y, N439K, and L452R at spike protein has increased 10%-92%. Evolutionary rate of the variants was 23.7 substitution per site per year. Substitution mutations E484K and N501Y had significant correlation with cases (*r* = .45, *r* = .23), fatalities (*r* = .15, *r* = .44) and growth rate R_0_ (*r* = .28, *r* = .54). This study will help to understand the genomic diversity, evolution and the impact of the variants on the outcome of the COVID-19 pandemic.

## Introduction

The ongoing pandemic, coronavirus disease-2019 (COVID-19) has been set out by a novel species of coronavirus namely, severe acute respiratory coronavirus-2 (SARS-CoV-2) of family *Coronaviridae* [1–4]. Among members of the genera *Betacoronavirus*, only SARS-CoV-2 has set out a severe pandemic [5–8]. On the second week of December 2019, the first confirmed case of SARS-CoV-2 was reported from Wuhan, China [1–3]. As of February 02, 2022, about 390 million cases and 6 million fatalities of COVID-19 have been confirmed from more than 219 countries and territories globally [9–11]. A sharp increase of COVID-19 cases and fatalities have been identified from October, 2020 globally. Four distinct waves of cases and fatalities have been traced after the onset of the pandemic [9–11].

Infected individuals are the main sources of transmission. Transmission of SARS-CoV-2 can occur via direct or indirect contact, droplets and fomites [1,3,12]. Viability of SARS-CoV-2 can range from 2 hours to 9 days in environment [12,13]. In symptomatic infection COVID-19 patients develop significant clinical manifestations of the respiratory system [14]. The incubation period of COVID-19 may vary from 2 to 14 days, but on an average it takes 4–7 days to develop the symptoms. Fever, chill, dry cough, sore throat and shortness of breath or difficulty in breathing are the most common clinical features of COVID-19 during the circulation of alpha and beta variants [14]. Symptoms such as loss of taste or smell, feelings of shaking, headache, rash, conjunctivitis, muscle pain, congestion or runny nose, discoloration of fingers or toes have been noticed during the circulation period of delta variants among significant number of COVID-19 patients [14]. In severe cases, patients develop symptoms like, shortness of breath, chest pain, loss of speech or movement, difficulty in breathing, acute respiratory syndrome, acute pneumonia, heart failure, kidney failure and failure of multiple organs [14].

SARS-CoV-2 is one of the largest spherical, enveloped RNA viruses that can infect human [15]. It is a single stranded, non-segmented, positive sense RNA virus. The size of the complete genome is ~30,000 bases in length [15–18]. At the upstream region, the genome contains a 5′ cap and at the downstream region a 3′ poly (A) tail. Ten open-reading frames (ORFs) namely, 1a, 1b, 3a, 3b, 6, 7a, 7b, 8a, 8b and 9b with four structural protein coding ORFs have been characterized in SARS-CoV-2 genome [16–18]. Among the ORFs, first two ORF namely, ORF-1a and ORF-1b occupy ~20,000 bases. The ORF-1ab (two-third of genome) mainly encodes for nonstructural (nsps) proteins [15–21]. About 16 nonstructural proteins namely, nsp1 to nsp16 have been characterized with defined functions. The ORFs including 10000 bases of the genome at 3′ end encodes four major structural proteins- spike (S), envelope (E), membrane (M) and nucleocapsid (N) [18]. The well-defined genome order of coronavirus is 5′ leader-UTR-replicase-S-E-M-N-3′ UTR-poly (A) [15–21].

Phylogenetic and evolutionary analysis revealed that SARS-CoV-2 genetically clusters with two bat-derived strains in subgenus *Sarbecovirus* (lineage B) [18]. At the whole genome level, about 96%, 91% and 82% identity have been detected between SARS-CoV-2 and bat coronavirus (BatCov RaTG13), between SARS-CoV-2 and Malaysian pangolin (*Manis javanica*) coronavirus, and between SARS-CoV-2 and SARS-CoV, respectively [2,15–21]. At protein level, the M protein of SARS-CoV-2 is 98.6%, 98.2% and 90% homologous with the M protein of bat SARS-CoV, pangolin SARS-CoV, and SARS-CoV, respectively [19–21]. The receptor-binding domains (RBDs) (position 331–524) of the spike glycoprotein from of SARS-CoV-2 and SARS-CoV share about 72% identity in amino acid sequence [19–21]. ACE2 sequence alignment reveals that dog, cat, pangolin and mink can become the potential host of SARS-CoV-2 [19–23]. Mutations including substitution point mutations, deletion and cluster mutations in RBD and nearby regions at spike glycoprotein have significant consequences in the virulence and disease outcome of COVID-19 [19–21].

Variants with cluster of substitution point mutations at spike protein have been identified to be involved with altered rate of transmission of COVID-19 [24–27]. After December, 2019, different variants including Alpha (B.1.1.7), Beta (B.1.351), Gamma (P.1), Delta (B.1.617.2), Lambda (C.37), Omicron (B.1.1.529), Epsilon (B.1.429), Zeta (P.2), Eta (B.1.525), Theta (P.3), Lota (B.1.526), Kappa (B.1.617.1) and Mu (B.1.621) have evolved. Among them, Alpha (B.1.1.7), Beta (B.1.351), Gamma (P.1), Delta (B.1.617.2), Omicron (B.1.1.529) have been designated as variants of concern (VOC) and Lambda (C.37) and Mu (B.1.621) as variants of interest (VOI) by WHO [26–35]. Numerous substitution point mutations at the spike protein have been identified to be involved with the evolution of these variants. Significant substitutions at spike included K417N, K417T, N440K, L452R, S477N, E484K, T478K, N501Y, D614G, P681R, P681H and others [28–38]. Circulation of Alpha, Beta, Gamma and Delta variants have increased incidence, hospitalization and fatality by 10% to 85%, while omicron variant has increased the incidence and lowered hospitalization [26,38].

Global Initiative on Sharing All Influenza Data (GISAID) and Nextstrain have suggested two different nomenclature systems for clade of COVID-19 [39,40]. According to GISAID, eleven clades namely, G, GH, GK, GV, GR, GRA, GRY, L, O, V, and S have been classified [39].

Evolution of variants with capability of escaping immunity will impose a global challenge in achieving sustainable public health goal [26,38]. More studies are needed to evaluate the available vaccine efficacy against newly evolved variants [41,42]. Otherwise, the global health burden of the ongoing COVID-19 will increase despite vaccination. This study is conducted to create a cumulative database on the circulation of COVID-19 variants globally. Integrated data on evolutionary divergence and mutational profile of SARS-CoV-2 is necessary to establish effective diagnosis, prevention and ultimate reduction of disease burden globally. Genomic analysis will provide detailed insights not only to prepare for potential waves but also to discover effective therapeutics and vaccines. The main aim of this study is to understand the origin and spread of COVID-19 variants during the pandemic. This study will create a basic guideline for future studies to understand the evolution of SARS-CoV-2.

## Materials and methods

### Data collection

Sequence data of COVID-19 were analyzed in this study. Sequence data were retrieved from two different databases. At first, sequence data were retrieved from GISAID (https://www.epicov.org/epi3/frontend#) and finally, from NCBI Virus (https://www.ncbi.nlm.nih.gov/labs/virus/vssi/) database [39,43]. Complete sequences of high coverage value were analyzed in this study. In the inclusion criteria, the sequence data collection and submission date were considered. The collection and submission date of the sequences ranged from December 01, 2020 to January 31, 2022. Sample collection date was considered as the reporting date of the genome. For the temporal and spatial transmission analysis of the variants, four time frames were determined. Each time period was consisted of three months namely, January 01- March 31, April 01- June 30, July 01- September 30 and October 01- December 31. For analyzing the regional variation data collection were made for all the time frames from 211 countries and territories in Africa, Americas, Asia, Europe and Oceania. A total of 7540558 whole genome sequences were analyzed during December 2019 from January 2022. Most of the sequences were collected from GISAID (7503558 sequences) followed by NCBI (37000 sequences). Data on COVID-19 cases, fatalities, growth rate, detection rate and vaccination were collected from different authorized local databases and global databases. Exclusion criteria included mutually common sequences of GISAID and NCBI databases.

### Data availability and analyses

The data used in this study were from Worldometer (https://www.worldometers.info/coronavirus/), Bing COVID-19 dashboard (https://www.bing.com/covid/), Johns Hopkins Coronavirus Resource Center (https://coronavirus.jhu.edu/map.html), UK governmental site (https://coronavirus.data.gov.uk/), GitHub COVID-19 database (https://github.com/CSSEGISandData/COVID-19), CDC COVID-19 Research Articles Downloadable Database (https://www.cdc.gov/library/researchguides/2019novelcoronavirus/researcharticles.html), World Health Organization Situation Report (https://www.who.int/emergencies/diseases/novel-coronavirus-2019/situation-reports), GISAID (https://www.epicov.org/epi3/frontend#), NCBI Virus (https://www.ncbi.nlm.nih.gov/sars-cov-2/), and Nextstrain (https://nextstrain.org/ncov/global) [39,40,43–45]. Extracted data from these websites were analyzed and rechecked for redundancy of the data. Data were collected and sorted according to the temporal and spatial distribution. Cluster and lineage definition were determined based on GISAID markers. Consistency and significance of the relationship of the data were determined by appropriate mutational analysis.

### Whole genome sequence analyses

Only full length sequences of whole genome between 29000 and 31000 bp were used in this study. Genome sequences from both NCBI and GISAID databases were analyzed. Multiple sequence alignment (MSA) was conducted using the BioEdit 7.2.6 software by using the ClustalW Multiple Alignment Algorithm [46–48]. The similarity matrix of these whole genome sequences was computed using the Maximum Composite Likelihood model. Each sequence was aligned to the reference sequence NC_045512.

### Phylogenetic analyses

Phylogenetic and evolutionary relationship analysis of COVID-19 were conducted using whole genome between 29000 base pairs and 31000 base pairs using the reference sequences Wuhan/WIV04/2019 by using the MEGA X software [46–48]. In this analysis mutations such as long substitutions, SNPs and indels were calculated. Trees were built using Maximum Composite Likelihood (MCL) method and genetic distance was calculated by Kimura-2-parameter (K2P) model [47,48]. Phylogenetic trees were generated with 1000 bootstrap replicates of the nucleotide alignment datasets. For phylogenetic tree building whole genome sequences from NCBI database were used [49]. Four hundred and eighty five sequences from Africa, 855 sequences from Asia, 624 sequences from Europe, 738 sequences from North America, 395 sequences from Oceania and 625 sequences from South America were used in this study to construct the phylogenetic trees, respectively. Another phylogenetic tree with 95% confidence interval was constructed for the determination of evolutionary distance among 22 published reference sequences of *Betacoronavirus* isolated from different animals.

### Mutational analyses

Mutational analysis of the whole genome were conducted in nucleotide and amino acid sequences. Mutations such as SNPs, indels, long substitutions, deletion or insertion of bases were considered in this study. We analyzed both the mutations persisting for long time and transitional mutations that appeared for certain period of time and were outnumbered by other mutations or disappeared globally. Multiple sequence alignment (MSA) was conducted by using ClustalW in MEGA X and NC_045512 were used as the reference sequences [47–50]. For conducting mutational analysis, whole genome from both databases were aligned.

### Statistical analyses

Appropriate statistical analysis was conducted to predict the correlation of the mutants with the outcome of the pandemic. In statistical analysis, *p* value less than .05 was counted as statistically significant and Pearson’s coefficient, *r* greater than 0 to +1 was considered as positive correlation between variables, while *r* value less than 0 to -1 was considered as negative correlation between variables. Standard deviation were calculated and 95% confidence intervals were applied for where applicable. The relationships among finite groups of significant mutants were determined by Venn diagram. Both the spatial and temporal distribution of the spike protein mutations and other significant mutations of structural and non-structural proteins were considered in the Venn diagram. Statistical analyses were conducted by using SPSS version 25 (IBM Inc., Armonk, NY), and STATA software, version 14 (StataCorp. 2015. Statistical Software: Release 14.0. College Station, TX: Stata Corporation).

## Results

### Temporal and spatial distribution of COVID-19 clades

**Fig 1:**
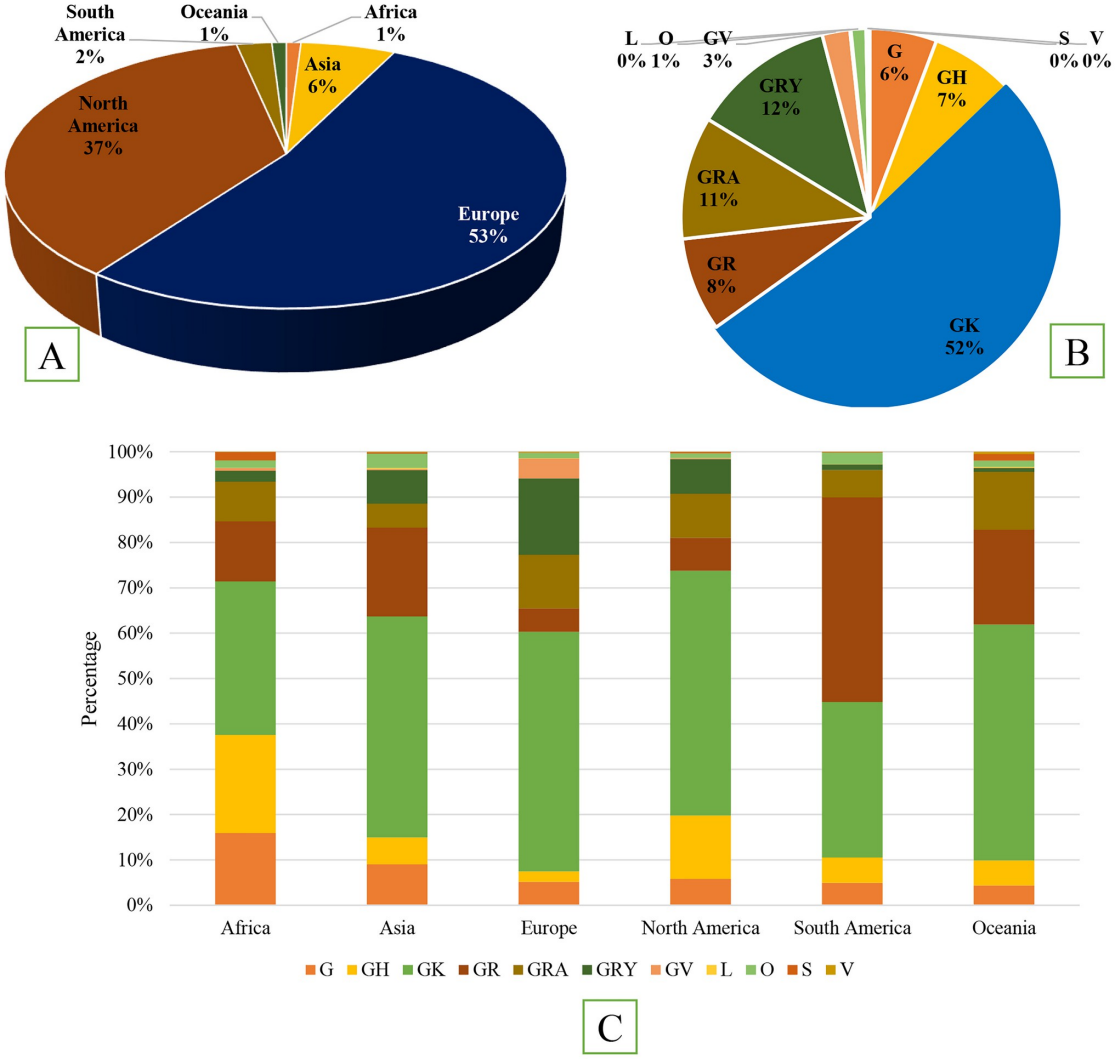
(A) Frequency distribution of total of 7540558 whole genome of SARS-CoV-2 among six continents. All of the whole genomes were collected and submitted to GISAID and NCBI during December, 2019 to January, 2022. Only genome sequences of high coverage of reference and complete genome were included in this analysis. (B) Frequency distribution of 7540558 whole genome of SARS-CoV-2 into eleven clades isolated during December, 2019 to January, 2022. (C) Distribution of eleven clades of 7540558 whole genome of SARS-CoV-2 in six continents.

Total 7540558 whole genome of high coverage and quality had been reported globally during December, 2019 to January, 2022. Most of the sequences have been reported form Europe (53%), followed by North America (37%), Asia (6%), South America (2%), Africa (1%) and Oceania (1%), respectively (Fig 1). Isolates were distributed into eleven clades determined according to the presence of different combination of markers in the genome. About 4.9% of the isolates were reported during December, 2019 to December, 2020. Among the clades, GK (52%) was the most predominant followed by GRY (12%), GRA (11%), GR (8%), GH (7%), G (6%), GV (3%), and O (1%), respectively (Fig 1). Isolates from clades including L, O, S and V had reduced significantly after July 01, 2020. Total 73654 whole genome from Africa were analyzed. In Africa, GK (34%) was the most prevalent clade followed by GH (22%) and G (16%) (Fig 1). About 493484 whole genome were included in Asia. Clade GK (47%), was most frequent followed by GR (20%), G (9%), and GRY (6%) in Asia. In Europe, North America and Oceania, GK (55%, 52% and 52%, respectively) was the predominant clade and in South America GR (45%) was the most predominant (Fig 1).

**Fig 2:**
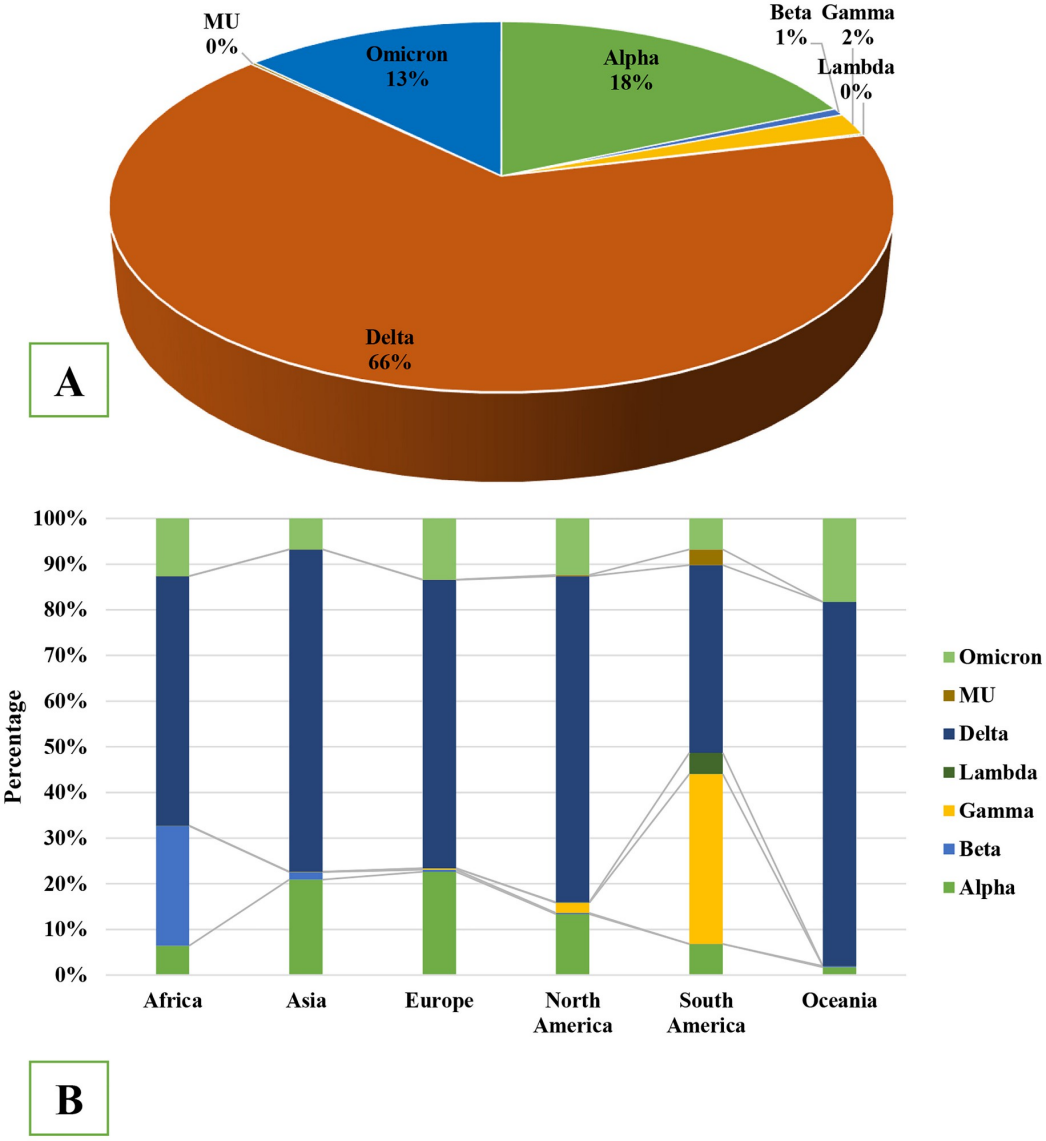
(A) Global frequency distribution of 7540558 whole genome of SARS-CoV-2 among variants of concerns identified during December, 2019 to January, 2022. (B) Percentage of variant of concerns of SARS-CoV-2 circulating throughout the six continents during the COVID-19 pandemic.

Among them, 49915 were isolated from Africa, 357658 from Asia, 3491173 from Europe, 2174115 from North America, 164572 from South America and 49616 from Oceania. Among VOC and VOI, seven variants were detected in significant frequency globally. Delta (66%) was the most prevalent variant followed by Alpha (18%), Omicron (13%), Gamma (2%) and Beta (1%), respectively. After the circulation of Delta variant, it became the most predominant in Africa (54%), Asia (71%), Europe (67%), North America (72%), Oceania (80%) and South America (40%) (Fig 2). In South America VOC gamma was detected in significant frequency (40%) and in Asia (20%), Europe (22%) and North America (15%) VOC alpha was isolated in significant frequency after VOC delta (Fig 2). After November, 2021, VOC omicron was detected in significantly higher frequencies in Africa, Europe, Oceania and North America.

During January 01, 2020 to December 31, 2020, SARS-CoV-2 had been detected from environmental samples and various animals including mink, dog, cat, lion, tiger and mouse (Table 1). Among 7540558 whole genomes, only 370000 were available during January to December, 2020. Circulation in various animals is an indication of a broad host range of SARS-CoV-2. Besides human, these animals can serve reservoirs and source of transmission.

**Table 1:** List of confirmed hosts of SARS-CoV-2 with the isolated regions during the first year of the COVID-19 pandemic, December, 2019 to December, 2020.

Host	Number of isolates	Continents (Country)
Human	369152	Africa, Americas, **Asia (China)**, Europe, Oceania
Environment	76	Americas, **Asia**, Europe
*Felis catus* (Cat)	15	Americas, Asia, **Europe**
Mink	4	**America (Canada)**
*Mus musculus* (House mouse)	1	**Asia (China)**
*Canis lupus familiaris* (Dog)	4	**Asia**, America, Europe
*Mustela lutreola* (European mink)	13	**Europe (Netharland)**
*Neovison vison* (American mink)	725	**Europe**
*Panthera leo*	4	**America (USA)**
*Panthera tigris jacksoni*	6	**America (USA)**
**Total**	370000	

Bold letter indicates the first isolation place.

### Spatial and temporal distribution of substitution point mutations of SARS-CoV-2

**Fig 3:**
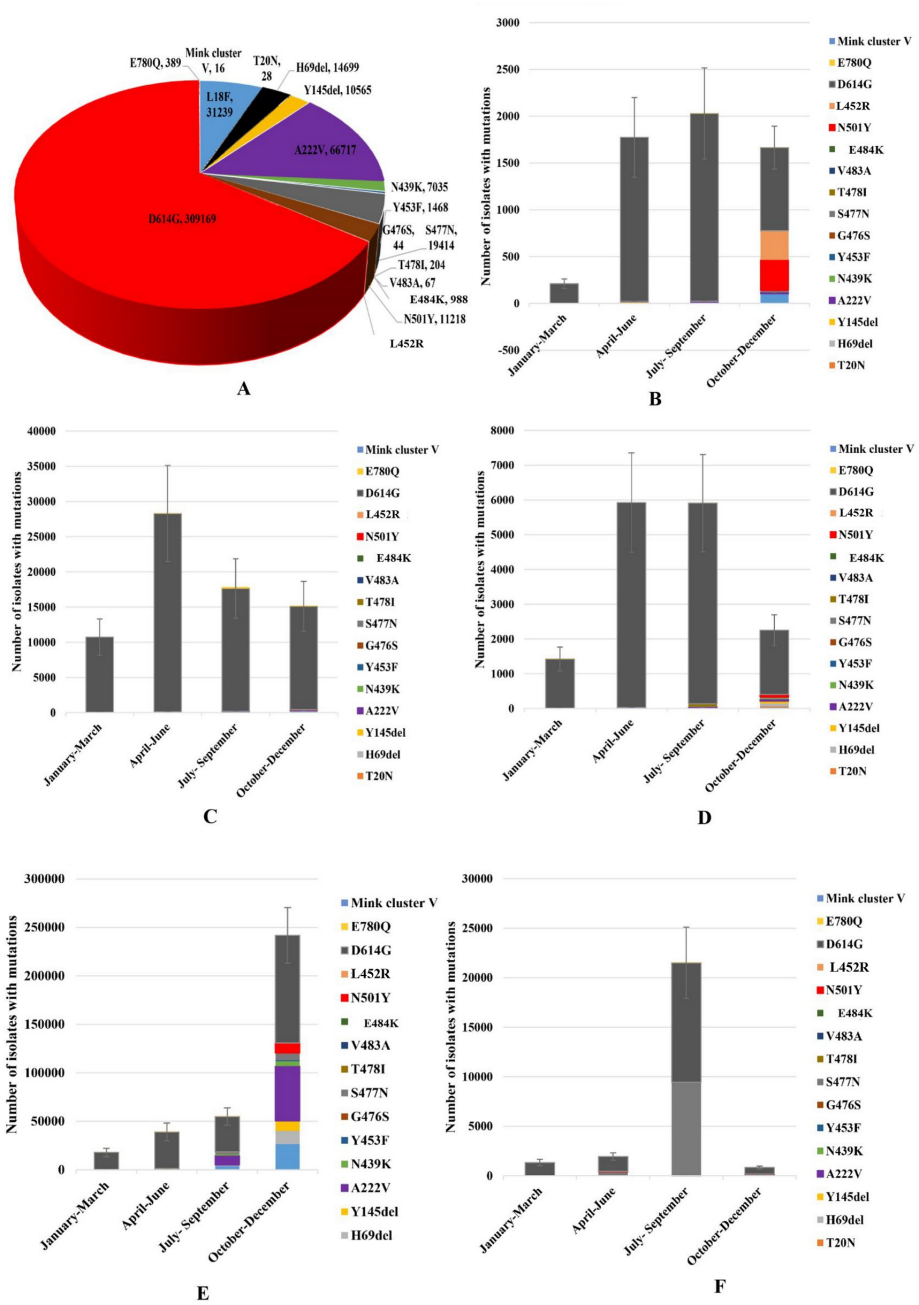
(A) Temporal and spatial distribution of substitution of frequency of point mutations at spike protein of SARS-CoV-2 worldwide during January, 2020 to December, 2020. (B-F) Continent-wise frequency distribution of the detected significant mutations at spike protein during January, 2020 to December, 2020 [(B) Africa, (C) America, (D) Asia, (E) Europe, and (F) Oceania, respectively]. Wuhan-Hu-1/2019 was used as reference sequence in this analysis.

**Fig 4:**
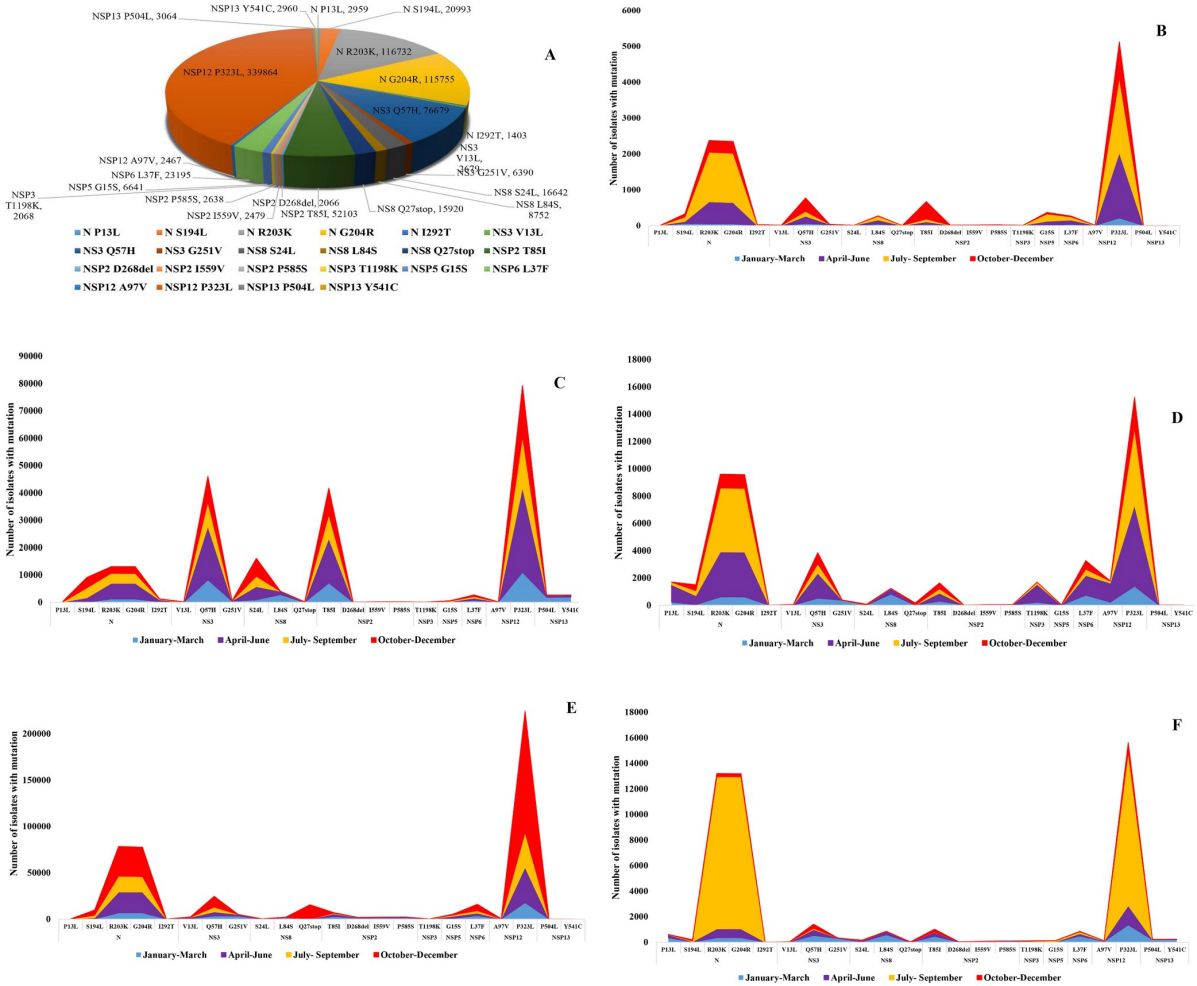
(A) Worldwide distribution of frequency of significant substitution point mutations at nucleocapsid and nonstructural protein of SARS-CoV-2 during January, 2020 to December, 2020. (B) Monthly report of substitution point mutations at nucleocapsid and nonstructural protein of SARS-CoV-2 in Africa and (C) Americas, (D) Asia, (E) Europe, and (F) Oceania during January 01 to December 31, 2020. Wuhan-Hu-1/2019 was used as reference sequence in this analysis.

About 17 significant mutations at spike protein were detected in this study. During January 01, 2020 to December 31, 2020, the most predominant mutation at spike protein was D614G (84%), followed by A222V (18%), L18F (8.5%) S477N (5%), H69del (4%) and N501Y (3%), respectively (Fig 3). Among these substitution point mutations, T20N (28), G476S (44), T478I (204) and V483A (67) were detected in very low frequency globally. The frequency of cluster mutations at RBD including, E484K and N501Y are increasing rapidly after first report. In Africa, among 80730 genome, E484K (8%) was predominant, followed by N501Y (5%), and L18F (2%), respectively (Fig 3 part B). About 100% of N501Y, and 99% E484K were detected during October 01- December 31 in Africa. In Americas, among 430521 genome, after D614G, E484K (21%) was the most significant, followed by L18F (15%), S477N (9%) and A222V (8%), respectively. The frequency of N501Y was about 3% in America (Fig 3 part C). In Asia, among 10435 genomes, H69del (22%) was the most prevalent, followed by Y145del (19%), A222V (17%), T478I (17%) and N501Y (17%), respectively. About 100% isolates with N501Y had been detected during October 01- December 31 in Asia (Fig 3 part D). In Europe, among 264896 whole genomes, A222V (47%) was the most significant followed by, L18F (22%), H69del (10%) and N501Y (8%), respectively (Fig 3 part E). The frequency of E484K and N501Y was about 99% during October 01- December 31, while E484K was circulating since March 2020 in Europe. In Oceania, among 23478 genomes, S477N (98%) was the most significant mutation. About 95% of S477N were detected during July 01- September 30 in Oceania. About 1% isolate was detected with N501Y while only one isolates with E484K was found in Oceania (Fig 3 part F). Among other structural and non-structural proteins, about 22 significant point mutations and deletions were detected in N, NS3, NS8, NSP2, NSP3, NSP5, NSP6, NSP12 and NSP13 globally. Further, NSP12 P323L (93%) was the most prevalent, followed by N R203K (46%), N G204R (46%), NS3 Q57H (22%) and NSP2 T85I (17%), respectively (Fig 4 part A). Of note, the frequency of substitution mutation P13L, I292T and V13L at N, D268del at NSP2, T1198K at NSP3, P504L and Y541C at NSP13 reduced during October 01- December 31 globally. A peak of frequency of mutations R203K and G204R at N protein and another peak of frequency of P323L at NSP12 were detected during July-September in Africa (Fig 4 part B). In Americas, one peak of frequency of Q57H at NS3, one peak of frequency of T85I at NSP2 and another one of P323L at NSP12 were confined in a period of three months, April to June 2020 (Fig 4 part C). In Asia and Oceania a peak of frequency of mutations R203K and G204R at N protein and another peak of frequency of P323L at NSP12 were detected during July-September (Fig 4 part D). In Europe, a peak of frequency of mutations S194L, R203K and G204R at N protein and another peak of P323L at NSP12 were found during October to December (Fig 4 part E). In Oceania, similar to Europe, a peak of S194L, R203K and G204R at N protein and another peak of P323L at NSP12 were confined during October to December (Fig 4 part F).

### Spatial and temporal distribution of prevalent point mutations in SARS-CoV-2 genome

**Fig 5:**
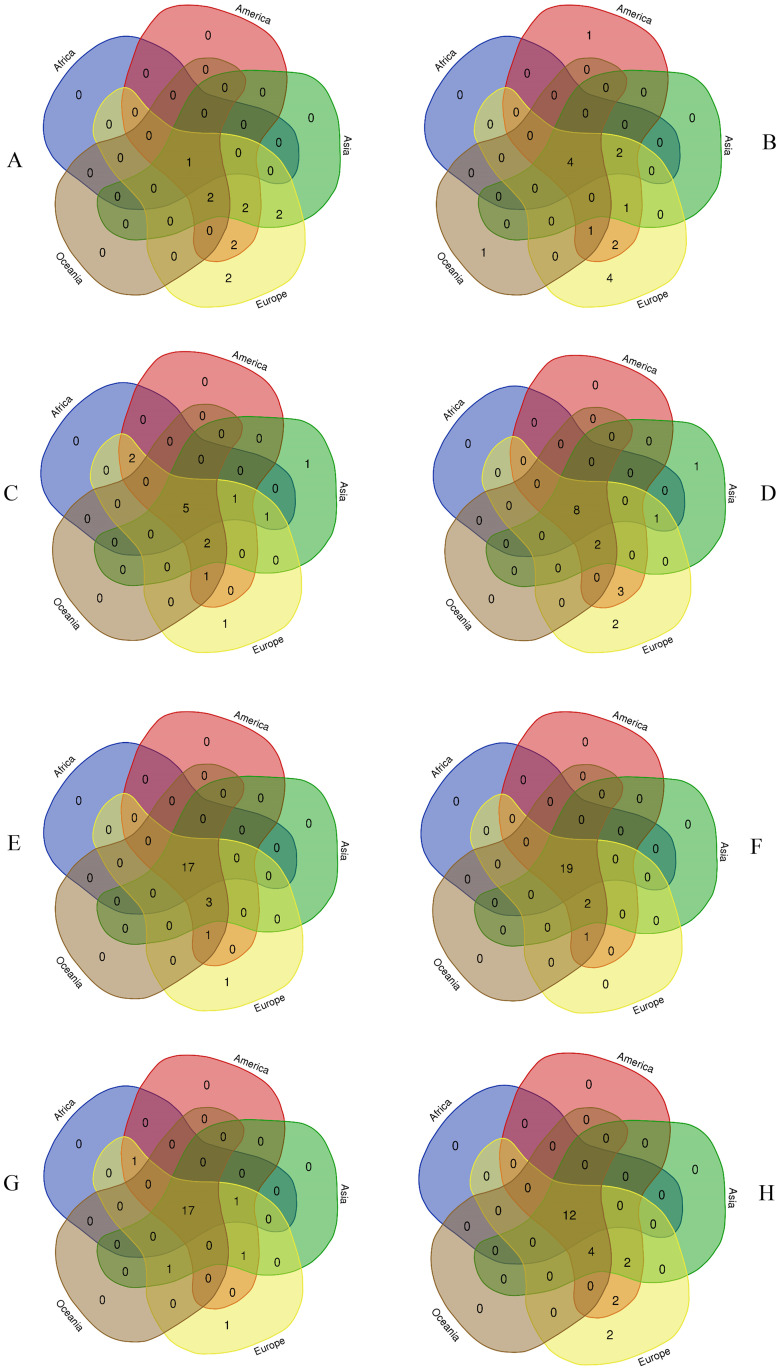
Venn diagram representing the incidence of significant point mutations at RBD and S1/S2 junction at spike protein of circulating SARS-CoV-2 in six continents during (A) January to June, 2020 (B) July to December, 2020 (C) January to June, 2021 and (D) July to December, 2021. The frequency of collective point mutations and common point mutations at S protein among the six continents increased significantly after June, 2020. Venn diagram representing the incidence of significant point mutations at nucleocapsid and nonstructural protein among circulating SARS-CoV-2 in Africa, Americas, Asia, Europe and Oceania during (E) January to June, 2020 (F) July to December, 2020 (G) January to June, 2021 and (H) July to December, 2021. Total of 7663308 whole genome of SARS-CoV-2 were analyzed. Wuhan-Hu-1/2019 was used as reference sequence in this analysis.

Total of 7540558 sequences were analyzed for detecting the presence and spread of substitution point mutations across the six continents. Origin of substitution mutations and deletions at RBD of spike protein increased and disseminated with high frequency globally during the pandemic. About 11 point mutations were detected at spike protein during January to June, 2020 globally (Fig 5 part A). Substitution D614G at S protein was circulating in all the continents at the beginning of the pandemic. In Europe, all 11 substitutions were circulating, followed by Asia (7 substitutions), Americas (7 substitutions), Oceania (3 substitutions), and Africa (1 substitution), respectively. The frequency of D614G at S protein in all the continents was 9% (1/11) during January to June, 2020.

During July to December, 2020, 16 significant point mutations were detected at S protein. Among them, H69del, L18F, D614G and S477N (25%, 4/16) were circulating in all the continents (Fig 5 part B). Europe was reported to be with most number of substitutions (14) followed by America (11) (Fig 5 part B). Though the total number of significant point mutations decreased, the frequency of common substitution point mutations and deletion (E780Q, H69del, L18F, D614G and A222V) increased to 36% (5/14) during January to June, 2021 (Fig 5 part C). During July to December, 2021, the total number of substitution and deletion has increased 3.3 times than the first six months of 2021. The frequency of common point mutations (E780Q, E484K, P681R, N501Y, N439K, D614G, L452R, and A222V) at S protein increased to 47% (8/17) during July-December, 2021 globally (Fig 5 part D). Among the 17 substitutions, most of them were circulating in Europe (94%), followed by America (76%) and Asia (70%).

Other substitutions and deletions at nonstructural and structural proteins were also analyzed. Total of 22 point mutations were detected during January to June, 2020 globally. Among them, 17 (77%) were circulating in six continents (Fig 5 part E). The highest number of substitution was found in Europe (22) followed by America (21) and Asia (20) during January to June, 2020. The number of common mutations circulating globally increased to 19 during July to December, 2020. The highest frequency (86%, 19/22) of common mutations and deletions (N P13L, N S194L, N R203K, N G204R, N I292T, NSP6 L37F, NSP2 D268del, NSP2 T85I, NSP2 I559V, NSP2 P585S, NSP3 T1198K, NS3 Q57H, NS3 V13L, NS3 G251V, NSP5 G15S, NS8 S24L, NS8 L84S, NSP12 P323L, and NSP12 A97V) were detected across six continents (Fig 5 part F). The number of common substitution and deletion mutations (N P13L, N R203K, N S194L, N G204R, NSP6 L37F, NSP2 T85I, NS3 Q57H, NS3 V13L, NSP5 G15S, NS8 Q27stop, NS8 L84S, and NSP12 P323L) reduced to 54% (12/22) from 77% (17/22) in during July to December, 2021 (Fig 5 part H). However, during July to December, 2021, the total number of significant point mutations circulating in Europe remained the same as the first six month of 2021.

### Probable origin and spread of variant of concerns and trends of global cases and vaccination

**Fig 6:**
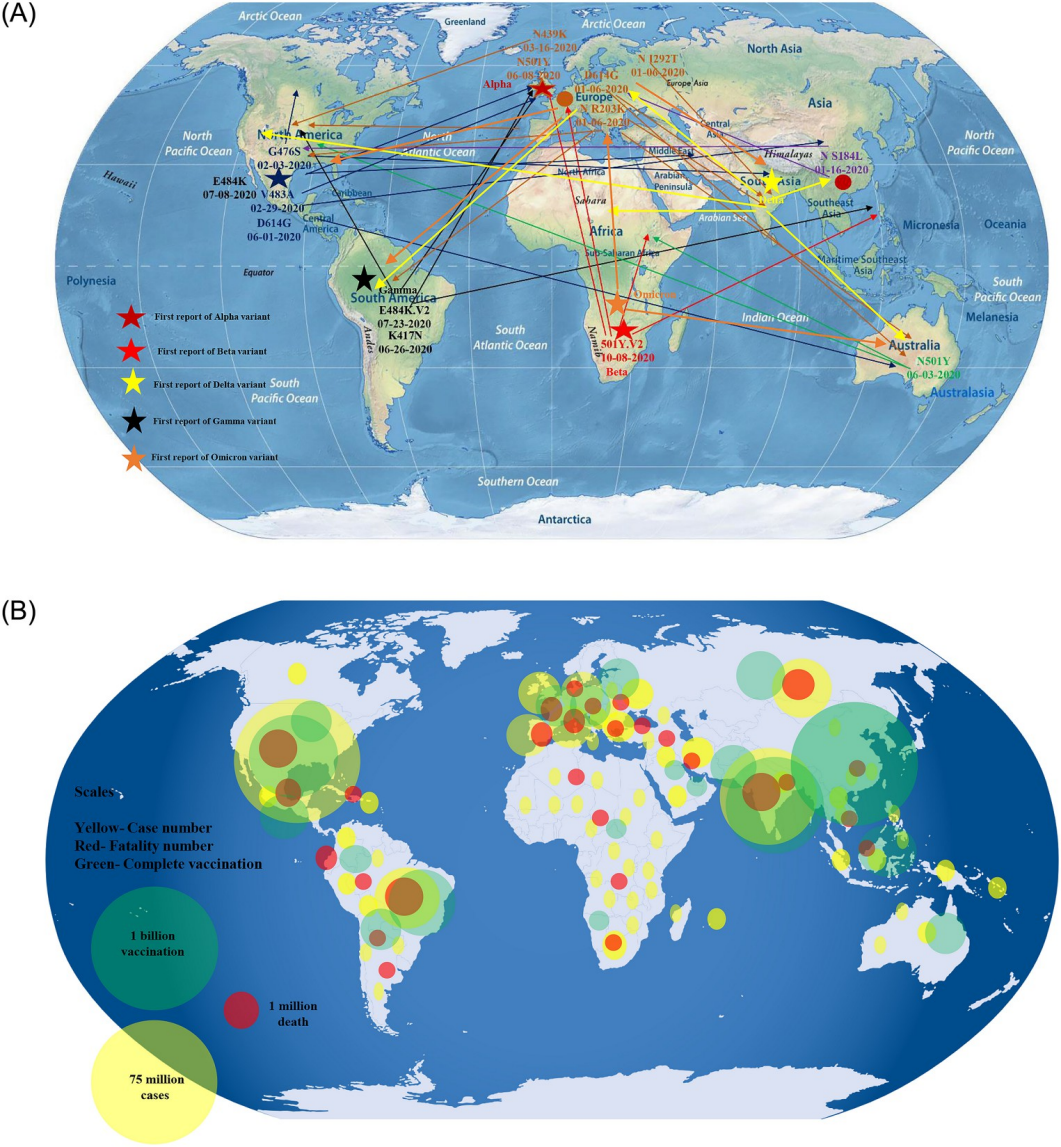
(A) Probable origin and global transmission history of significant variants of concerns of SARS-CoV-2 with altered transmission rate, fatality rate and detection rate during the COVID-19 pandemic. During December, 2019 to January, 2022 about 7540558 whole genome of SARS-CoV-2 were traced worldwide. Four variants of concern including beta, gamma, delta and omicron with their probable transmission history were indicated by four different color lines. Star and circle indicated probable origin places. (B) Worldwide distribution of cumulative case, fatality and complete vaccination number of COVID-19 till 31 January, 2022.

Total 7540558 sequences were analyzed during December 2019 to January 2022. After acquiring point mutations and successful transmission from human to human different variants of COVID-19 have prevailed worldwide. Depending on the presence of certain point mutations at spike protein, Alpha (B.1.1.7), Beta (B.1.351), Gamma (P.1), Delta (B.1.617.2), Omicron (B.1.1.529) have been identified as variants of concern by WHO globally. Before defining any variants, significant mutations at RBD and spike protein clustered and transmitted. Among the significant mutations N501Y, K417N, N439K, L452R, T478K and E484K were found to be present in VOCs (Fig 6A). Most of the VOC (80%, 4/5) were first identified during May, 2020 to November, 2020. A probable tracing and sequence analysis of available genomic data from GISAID and NCBI indicated that VOC Beta (B.1.351) was first circulating in South Africa during April to May, 2020 followed by VOC Alpha (B.1.1.7) in UK during August to September, 2020, VOC Delta (B.1.617.2) in India during August to November, 2020, VOC Gamma (P.1) in Brazil during August to November, 2020 and VOC Omicron (B.1.1.529) in South Africa during July to November, 2021 (Fig 6A). Point mutation D614G was first reported during January to March, 2020.

Reported cases of COVID-19 have increased to more than 400 million worldwide. USA has reported the most number of cases (20%), followed by India (10%), Brazil (6.8%), France (5%), UK (4.5%), Russia (3.3%), Turkey (3%), Italy (2.9%), Germany (2.9%) and Spain (2.5%), respectively (Fig 6B). Over 1 million deaths have been documented in Asia, Americas and Europe. Vaccination against COVID-19 has begun worldwide and about 60% of the world population has received at least one jab of vaccine. Most of the doses of vaccine have been administered in Asia, followed by North America, Europe and South America (Fig 6B). The cases and fatalities of COVID-19 have increased significantly after the circulation of VOC Delta (B.1.617.2) across the globe. After the circulation of VOC Omicron (B.1.1.529), large number of vaccinated population have been infected with COVID-19, but frequency of hospitalization and mortality have reduced comparatively.

### Phylogenomic and evolutionary analysis of SARS-CoV-2

**Fig 7:**
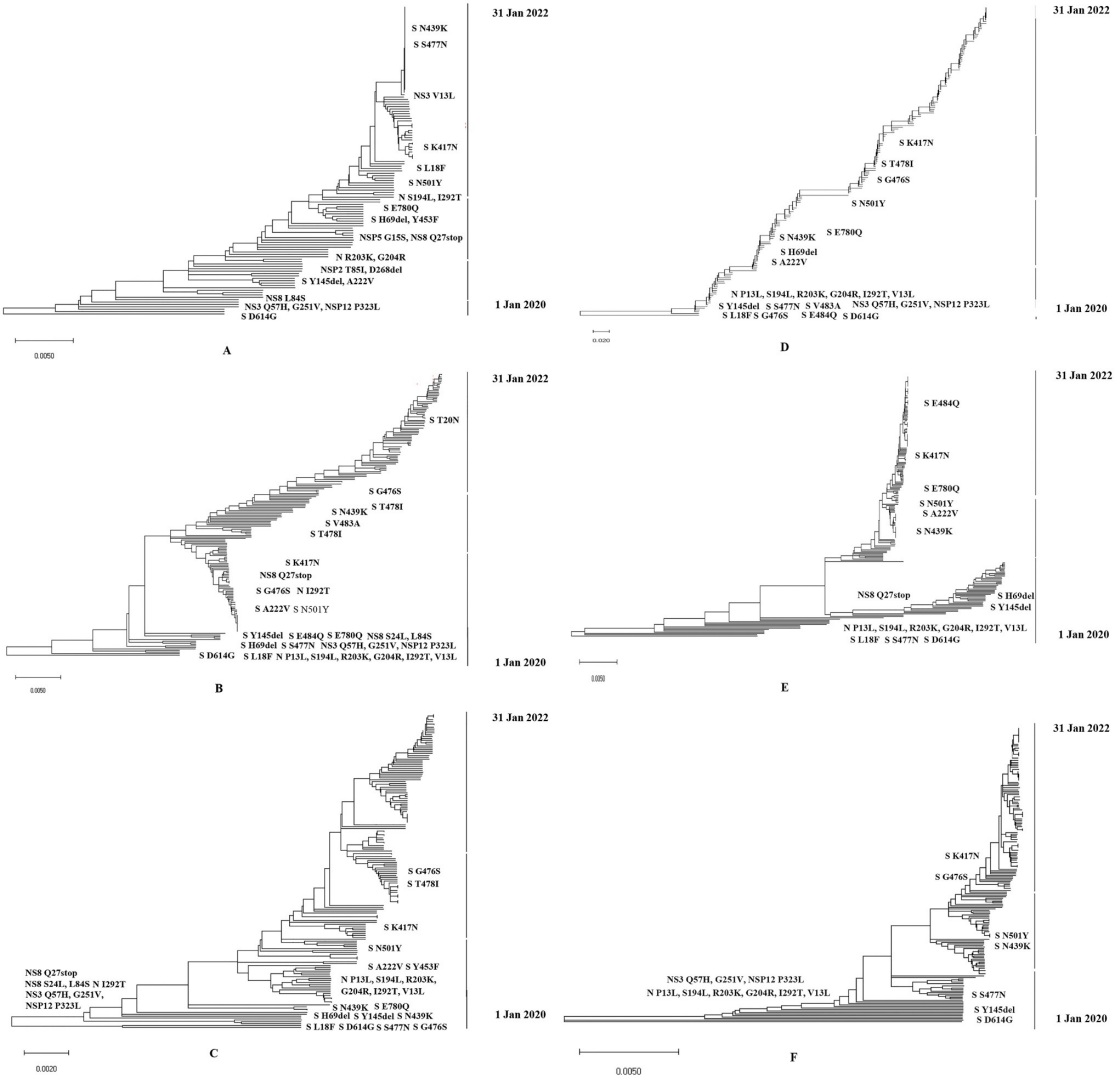
Phylogenomic tree of the whole genome of SARS-CoV-2 including the representative genomes containing significant point mutations. Whole genome with high coverage were selected for every continents. Every phylogenomic tree included at least 10 or more sample sequences collected in every month after December, 2019 to January 2022 in every continents. Reference SARS-CoV-2 strains were selected from NCBI. Trees were built by using Maximum Composite Likelihood (MCL) method and genetic distance was calculated by Kimura-2-parameter model. Phylogenomic trees were generated with 1000 bootstrap replicates of the nucleotide alignment datasets. Six trees represented six continents including (A) Africa (485 whole genomes), (B) Asia (855 whole genomes), (C) Europe (624 whole genomes), (D) North America (738 whole genomes), (E) Oceania (395 whole genomes), and (F) South America (625 whole genomes), respectively. Wuhan-Hu-1/2019 was used as reference sequence in this analysis.

Phylogenomic analysis of whole genome sequences were conducted to reveal the evolutionary relationship and changes in the circulating variants in six continents during December, 2019 to January 2022. In Africa, the variants containing multiple persistent substitution point and deletion mutations were circulating from the first quarter of 2020. The variants with multiple significant mutations at RBD and S1/S2 junction continued to evolve and Alpha (B.1.1.7), Beta (B.1.351), Gamma (P.1), Delta (B.1.617.2) started to circulate after September, 2020 (Fig 7 part A). During the early three months of the pandemic, sequenced isolates contained S D614G S Y145del, S A222V, N G204R and they were closely related with isolates in Asia, Europe and America (Fig 7 part A). After June, 2020, isolates in Africa were circulating with S N501Y, S H69del, S L18F, S K417N and S E484K point mutations. In Asia, isolates acquired mutations more rapidly than Africa. Most of the isolates in Asia were closely related with isolates in Europe and Americas. Significant mutations at spike protein and nucleocapsid were documented during January-June, 2020 (Fig 7 part B). Four distinct clusters of variants were detected in Europe. Isolates circulating during January to June 2020 contained S D614G, N501Y, E484K, V483A N439K H69del Y453F and many other point mutations at spike protein (Fig 7 part C). Isolates in Europe were closely related with isolates of China, India, South Africa and Indonesia. In Europe, most of the variants circulating during January- March evolved into isolates containing more mutations in RBD and other part of spike protein (Fig 7 part C). In North America, the evolution of the variants occurred during the first two quarters of 2020 with high frequency. Variants containing N501Y was circulating in North America during April-June, 2020 (Fig 7 part D). Isolates of North America were closely related with the reference sequences of Europe and Asia. In Oceania, two clusters of isolates were identified, one containing the variants with H69del and Y145del at spike proteins, other cluster contained the point mutations S N501Y and S E484Q (Fig 7 part E). Isolates in Oceania were closely related with reference sequences of Asia and Europe. In South America, about four clusters of the isolates were detected (Fig 7 part F). Significant cluster mutations at spike protein evolved during March to September, 2020 in South America. Isolates were highly similar with reference sequences of Asia and Europe.

**Fig 8:**
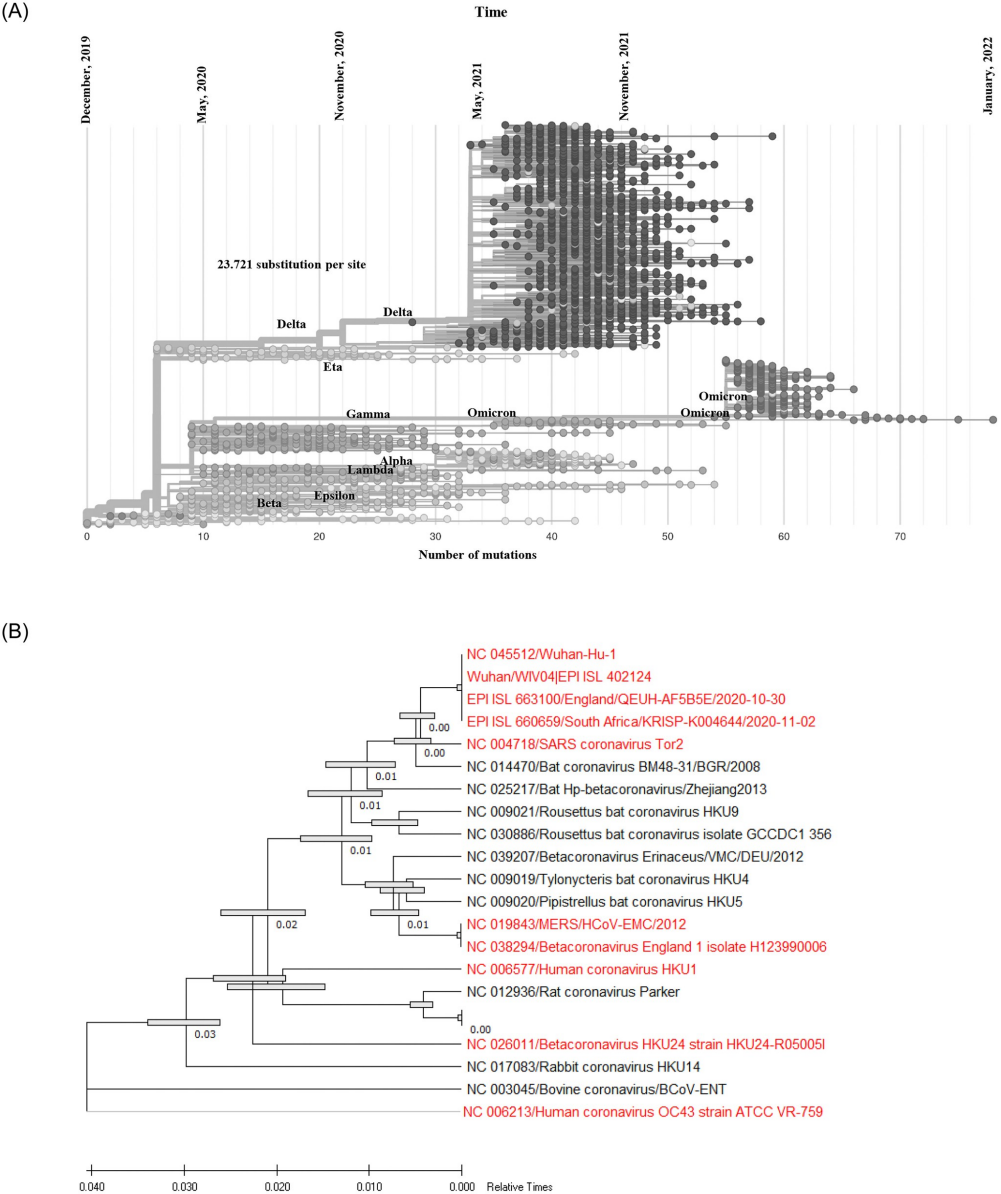
(A) Phylogenomic tree of 3055 representative whole genomes of SARS-CoV-2 isolated during December 2019 to January 31, 2022 representing the diversity and evolution of different variants. The tree is rooted relative to early samples from Wuhan, China. Nucleotide substitution rate of 8 × 10^−4^ subs per site per year was represented by single temporal resolution. Mutational frequency was calculated by using previous model (Obermeyer et al). The lower scale represented number of mutations and the upper scale indicated timeline of evolution. Wuhan-Hu-1/2019 was used as reference sequence in this analysis. (B) Phylogenetic tree of the whole genome of available reference sequences of coronaviruses isolated from different animals and human collected from NCBI. The tree was constructed using whole genome of novel coronaviruses. The tree was built by using the Maximum Composite Likelihood (MCL) method and genetic distance was calculated by Kimura-2-parameter model. Phylogenetic tree was generated with 1000 bootstrap replicates of the nucleotide alignment datasets. The scale indicates nucleotide substitutions, SNPs and indels per position. The bar in the branch indicated 95% confidence intervals. Red colored references indicated human coronaviruses. Total of 21 whole genome from 8 different animals were included in this analysis.

Evolutionary tree on the origin and spread of VOC and VOI revealed a comparative output globally. During January to June, 2020, isolates were acquiring point mutations in the genome and VOC Beta evolved with approximately 15–20 mutations at spike protein. A distinct cluster were separated from isolates of VOC Beta during March, 2020 evolving into VOC Delta with 15 to 20 point mutations at spike protein. Another distinct variant, Eta evolved from the same isolates of VOC Delta with about 20 mutations. VOC Delta were separated into four distinct sub variants and continued to transmit rapidly. Numerous isolates of VOC Delta have acquired 30 to 60 point mutations at spike protein. Isolates closely related to VOC Beta evolved into variant Epsilon. VOC Alpha and Lambda were closely related and evolved during the same period during the pandemic. Another distinct cluster of isolates evolved into VOC Gamma and VOC Omicron. VOC Omicron acquired 25 to 85 substitution point mutations at spike protein. Till January 2022, three sub variants of VOC Omicron has evolved (Fig 8A).

Origin and evolution of SARS-CoV-2 were analyzed (Fig 8B). The most recent descendants of human coronaviruses namely, NC_045512, Wuhan/WIV04/2019, and all the VOC were closely related with SARS coronavirus Tor2, and these five human coronaviruses were evolutionarily closely linked with Bat coronavirus BM48-31/BGR/2008 (Fig 8B).

### Correlation analysis of SARS-CoV-2 isolates with mutations with cases, fatalities, growth rate and detection rate

Significant changes in case number, fatality number, case fatality rate, transmission rate and detection rate had been detected with the evolution of new variants containing multiple mutations at the RBD and several site of spike protein controlling the interaction with epitopes of human hosts antibodies. The Pearson’s Correlation coefficient was determined among these variables. For conducting the analysis, samples were collected from four point of periods during January 01, 2020 to December 31, 2022. About 16 substitution point mutations and deletions at spike proteins were analyzed for the correlation with the outcome of the COVID-19 pandemic. Deletion mutation Y145del had the highest correlation (*r* = .8, *p* = .001) with the cases. Substitution mutations E484K, and N501Y had significant correlation with cases (*r* = .45, *r* = .23, *r* = .46, *p* = .001, *p* = .05, *p* = .003), fatalities (*r* = .15, *r* = .44, *r* = .87, *p* = < .0001, *p* = .0002, *p* = .005) and growth rate R_0_ (*r* = .28, *r* = .54, *r* = .49, *p* = .2, *p* = < .00001, *p* = < .00001) of the COVID-19 pandemic. Fatalities of COVID-19 had highest correlation with D614G (*r* = .9, *p* = < .001), followed by Y145del (*r* = .6, *p* = .006), respectively. With the detection rate of cases N501Y was negatively related (*r* = -.69, *p* = .001), E484K had minimum association (*r* = .0004, *p* = .001, *r* = .04, *p* = .0001) with the detection rate (Table 2).

**Table 2:** Pearson correlation coefficient between specific mutations of SARS-CoV-2 spike protein and COVID-19 cases, fatalities, transmission rate (R_0_), and detection rate.

Point mutation	Variables							
	Cases		Fatalities		Growth rate, R_0_		Detection rate	
	*p*	*r*	*p*	*r*	*p*	*r*	*p*	*r*
**L18F**	.003	.49	< .005	.24	.01	-.5	.2	.2
**T20N**	.006	.68	.01	-.21	.4	.01	.001	.03
**H69del**	.0001	.02	< .0005	.19	.001	.24	.001	.14
**Y145del**	.001	.80	.006	< .61	.05	.013	.1	.18
**A222V**	.001	.23	.001	.58	.001	.65	.3	.27
**N439K**	< .0005	.11	< .0001	.29	.002	.08	.001	.47
**Y453F**	.001	.45	< .001	.004	.001	.21	.001	.57
**G476S**	.007	.004	.005	.0001	.4	.05	.02	.68
**S477N**	.3	.19	.001	.34	.5	.07	.03	.004
**T478I**	.005	.02	< .00001	.17	.0001	.2	.004	.002
**V483A**	.003	.3	.5	.006	.001	-.19	.001	.24
**E484K**	.001	.45	< .0001	.15	.2	.28	.0001	.04
**N501Y**	.05	.23	.0002	.44	< .00001	.54	.001	-.69
**501Y.V2**	.003	.46	.005	.87	< .00001	.49	.001	.0004
**D614G**	.0001	.17	< .001	.9	< .000001	.74	.2	.57
**E780Q**	.03	.49	.07	.004	.005	.04	.07	.11

*P* value < .05 is considered statistically significant and positive *r* value of equal or less than 1 indicate significant correlation of variables.

COVID-19 transmission rate (R_0_), specific mutations of SARS-CoV-2 spike protein and COVID-19 and detection rate. *P* value < .05 is considered statistically significant and positive *r* value of equal or less than 1 indicate significant correlation of variables.

Other substitution point mutations and deletions at non-structural and structural proteins were also significantly associated with the outcome of the pandemic. Substitution mutation at NSP13 P504L had the most significant association (*r* = .9, *p* = .01) with the increase of cases, followed by NSP3 T1198K (*r* = .72, *p* = .001) and NSP13 Y541C (*r* = .61, *p* = < .0001), respectively. Substitution mutation at NS8 S24L (*r* = .8, *p* = .004) had the highest correlation with fatalities of COVID-19, NSP13 P504L were strongly correlated (*r* = .8, *p* = < .001) with the growth rate of COVID-19 and N S194L had significant association (*r* = .9, *p* = .1) with detection rate (Table 3).

**Table 3:** Pearson correlation coefficient between specific mutations of SARS-CoV-2 proteins and COVID-19 cases, fatalities, transmission rate (R_0_), specific mutations and detection rate.

Point mutation sites		Outcome of COVID-19 pandemic							
		Cases		Fatalities		Growth rate, R_0_		Detection rate	
		*p*	*r*	*p*	*r*	*p*	*r*	*p*	*r*
**N**	**P13L**	.2	-.4	.0001	.2	.01	-.8	.9	.3
	**S194L**	.4	.07	< .005	.001	.6	.2	.1	.9
	**R203K**	.003	.24	.06	.05	.7	.078	.7	.01
	**G204R**	< .0001	.004	< .005	.11	.005	.35	.0001	.32
	**I292T**	.5	.0007	.001	-.1	.001	.14	.001	.01
**NS3**	**V13L**	.0001	.01	.6	.6	.5	.4	.6	.61
	**Q57H**	.005	.14	.1	.07	.1	.75	.7	.23
	**G251V**	< .0001	.1	< .005	.43	.7	.68	.01	.27
**NS8**	**S24L**	.01	.41	.004	.8	.18	.41	.005	.17
	**L84S**	.1	-.01	.01	.07	.01	.7	.004	.3
	**Q27stop**	.07	-.4	.5	.1	.8	.4	.07	.8
**NSP2**	**T85I**	.001	.01	.7	-.4	.001	.14	.05	.1
	**D268del**	.3	.24	.01	.2	.9	.4	.001	.02
	**I559V**	.4	.01	.03	.18	.01	.07	.01	.1
**NSP2**	**P585S**	.006	.41	.001	.34	.005	.28	.42	.04
**NSP3**	**T1198K**	.001	.72	.04	.17	.01	.1	.001	.01
**NSP5**	**G15S**	.02	-.3	< .001	.28	.001	.45	< .0001	.07
**NSP6**	**L37F**	.5	.09	.9	.09	.05	-.08	.001	.21
**NSP12**	**A97V**	.1	.15	< .001	.4	.6	.01	.0001	.3
	**P323L**	.3	.2	.009	-.4	< .001	.59	.001	-.19
**NSP13**	**P504L**	.01	.9	.0001	.67	< .001	.8	.001	.06
	**Y541C**	< .0001	.61	< .00001	.14	< .0005	.3	.2	.01

*P* value < .05 is considered statistically significant and positive *r* value of equal or less than 1 indicate significant correlation of variables.

## Discussion

The COVID-19 pandemic is growing faster than before [9–11]. As the number of genome sequencing and analysis have been increasing, the diversity of SARS-CoV-2 variants is getting exposed [25,27,28,31,36,37,51]. We detected that about 53% of the sequence data in GISAID were from Europe followed by North America (37%), but in the NCBI database about 60% of the genomic data were from the USA only [39,43]. In comparison with other RNA viruses like influenza virus, the mutational events of SARS-CoV-2 is moderate [28,38]. However, different variants with modified immunogenic properties have already evolved. Clusters of substitution point mutations at RBD region and S1/S2 junction of spike protein are involved in altered epitope characteristics [20,22,28,49]. The temporal and spatial distribution of mutational events that occurred during the first two years of the pandemic were significant. The frequency of sequencing has increased after October 01, 2020. At the end of 2020 and the first two months of 2021 the diversity of variants with large number of substitution point mutations and deletions at the antibody binding protein (spike protein) has increased. The distribution analysis of clade revealed that GK (52%) was the most predominant followed by GRY (12%), GRA (11%), and GR (8%), respectively. Clade GR separated into GRY and GRA after first quarter of 2021 and their circulation remained relatively high after GK. The frequency of GR and GH increased significantly during July to December, 2020 in Africa, America, Asia and Oceania. However, GV became the most predominant clade during the last six months of 2020 in Europe. In Asia, Europe, and North America the frequency of GK clade was more than 50% during March, 2021 to December, 2021. The findings are similar with the data of GISAID [39]. This study supports that mutations of the virus are increasing with time throughout the genome. Through evolutionary changes acquisition of cluster of substitutions at RBD have significantly influenced the transmission rate and determining disease outcomes. Precise and early sequencing of circulating variants could play significant roles in reducing the rapid spread and large death of COVID-19.

Mutations at both structural and non-structural protein regions of the genome of COVID-19 have been detected globally [28,49]. Variants with mutations namely, SNPs, indels and deletions at 5′ UTR, NSP2, NSP3 (papain like protease), NSP6 (replicase nonstructural protein), NSP12 (RdRp) NSP13 (helicase), N, spike protein and 3′ UTR have been detected [49,52–55]. These mutations are involved in altered or changed interaction with antibody, replication efficiency, autophagy strategy, peptide processing capability, and proof reading mechanism during duplication and transmission of the virus [28,33,36–38,56]. VOC Delta and VOC Omicron have transmitted about 1.5 to 2 times faster than reference isolates [41]. During the circulation of VOC Delta, both the cases and fatalities increased significantly. Among many probabilities, lack of enough vaccination and the increased capability of transmission of VOC Delta might have influenced to develop a certain peak [57]. However, our study suggested that after vaccination of about 50% of world population, VOC Omicron have become predominant, which may be due to the altered transmission capability and reduction of practicing preventive measures of common people. Development of severe health conditions, hospitalizations and fatalities have reduced in certain countries with high case number of VOC Omicron, which might be due to the presence of certain immunity by vaccination or previous infection. These findings are supported by existing and growing data and previous studies [9,28,38,57].

Among different mutation sites, the most significant is the spike protein [28,49]. More than 80 substitution mutations and deletions have been reported in the spike protein globally. The significant mutations at spike protein were L452R, followed by E484K, D614G, A222V, L18F, S477N, H69del, and N501Y, respectively worldwide. The frequency of cluster mutations at RBD, along with L452R, and E484K is growing with high frequency in Africa, Americas and Europe. The accumulation of substitution and deletion mutations at immunogenic regions, 452, 484, and 501 of spike interrupt with immune-neutralization and involved with escape from immunity. Further, presence of substitution mutations only at 484 namely E484K have been found to reduce immune-neutralization also [26,33]. Further, variants with these mutations have retained the capability to bind with ACE2 receptor of host cells and in some variants the binding has become more effective. Among other structural and nonstructural proteins the frequency of substitution and deletion mutations varied in different continents. The diversity and frequency of mutations were significant in NSP12 (RdRp), NSP13 (helicase) and nucleocapsid proteins. Deletion of bases at 5′UTR (1–265) and 3′UTR (29675–29903) regions also increased during October 2020 to December, 2021. The findings of this study are in good agreement with previous studies [52–54]. In the phylogenomic analysis we detected the evolutionary relationship of the variants globally. The trees revealed that genomic diversity of SARS-CoV-2 has increased and different variants have clustered distinctly in six continents. The rate of substitution per site has increased to about 23.7, which reveals significant genomic diversity of the virus. This study reported that isolates with about 80 mutations per site have evolved within these two years, 2020 to 2021. Significant relation was found between VOC Beta and Epsilon; VOC Alpha and Lambda; VOC Gamma and VOC Omicron; VOC Delta and Eta. These findings are in good agreement with the databases and previous studies [44,49,58,59]. The frequency of common substitutions and deletions at RBD of spike protein has increased significantly during October to December and continued to increase faster in 2021 globally. These statements about substitution and deletion mutations are similar with the previous studies in Europe, America, Africa and Asia, but the frequency of mutations were detected significantly high in this study [28,49]. In the correlation analysis significant association of the E484K, and N501Y were detected with COVID-19 cases, fatalities and growth rate, but N501Y was negatively correlated with the detection rate. This is one of the first correlation analyses of COVID-19 pandemic with the variants containing mutations.

Findings from this study can be implemented to generate preliminary database on whole genome of COVID-19. Previous studies have included low number of whole genomes and partial analysis of the available data. However, this study has included all the available genome sequences to create a deep understanding of the evolutionary dynamics of the virus. Findings from this study will contribute in accurate tracing of the variants and future evolution of any other mutants of the virus globally.

This study will contribute in accurate diagnosis and detection of the highest possible variations of the genome. Determination of the regions with greater mutations will impact both the primer based molecular detection and immunological diagnosis [60]. The genomic changes in the primer binding regions affect both detection and diagnosis of SARS-CoV-2. Findings of this study will allow to design more universal primer for the molecular detection of the circulating mutants. Further, changes in the antigenic regions in the genome will also alter the specificity and sensitivity of rapid detection by immunological testing [60]. Inclusion of highest possible number of genome with mutations in the antigenic regions will also contribute to develop more accurate immunological testing methods.

Vaccine development and evaluation of effectiveness of vaccines against the emerging variants will become more convenient by using the findings in this study [41,42]. Moreover, worldwide analysis on the distribution of the mutations, variants and vaccination will contribute in understanding the pandemic and ways to prevent the transmission more effectively. Previous studies have represented partial data. Findings of this study will provide integrated baseline genomic database to understand the spread of variants with significant mutations and their impact on the vaccination worldwide. Further, effective antiviral designing and evaluation of effectiveness against all the variants with significant mutations also require genomic information. This study will provide improved and collective information in developing antivirals.

Researchers, and health professionals can get guideline information on the circulating variants from this study and use them for better preventive measures and management of the pandemic. For other infectious RNA virus including dengue virus, chikungunya virus and hepatitis virus outbreak, researchers and scientists can follow this study to create an integrated genomic database. Findings of this study will assist in public health practices in reducing the health burden by providing the necessary data on both the spread of the VOC of SARS-CoV-2 and vaccination. Further, policy makers can also use the collective information to implement effective policies in reducing the transmission and death related to COVID-19.

However, this study has few limitations like all the genomic data used in this study are from secondary sources which in future studies should be used from primary analysis to create more accurate data on the genomic sequences. The main strength of this study is the analysis of large number of available genome from most of the countries globally. Further, this study is strengthened by the inclusion of only full genome. Another major strength of this study is the evaluation of the annotation and alignment of the genomes by using different possible methods.

In future, more studies can be conducted to reveal the exact diversity of SARS-CoV-2. More and more genome sequencing should be done in Africa, Asia and South America. This study will provide crucial understandings of the diversity of SARS-CoV-2 evolved and circulating during the pandemic. High diversity of variants of SARS-CoV-2 has been detected in this study. Mutations in the RBD’s domains have significantly changed the transmission rate and severity of the disease. Further, different VOC has different impact on the health of people depending on the regions of origin and transmission. Vaccination and rapid circulation of less deadlier variants are associated with reduced mortality of COVID-19 globally. This study will provide a comprehensive insight of the origin, evolution, and diversity of circulating SARS-CoV-2 and their relationships with cases, fatalities, transmission rate and detection rate globally.
